# Obstructive sleep apnea and multimorbidity

**DOI:** 10.1186/1471-2466-12-60

**Published:** 2012-09-24

**Authors:** Laurence Robichaud-Hallé, Michel Beaudry, Martin Fortin

**Affiliations:** 1 Université de Sherbrooke, Québec, Canada; 2 Centre de santé et de services sociaux de Chicoutimi, Saguenay, Québec, Canada; 3 Département de médecine de famille, Université de Sherbrooke, 305, St-Vallier, Chicoutimi, Québec G7H 5H6, Canada

**Keywords:** Obstructive sleep apnea, Multimorbidity, Disease Burden Morbidity Assessment, Chronic disease, Severity

## Abstract

**Background:**

Obstructive sleep apnea (OSA) is becoming increasingly prevalent in North America and has been described in association with specific chronic diseases, particularly cardiovascular diseases. In primary care, where the prevalence of co-occurring chronic conditions is very high, the potential association with OSA is unknown. The purpose of this study was to explore the association between OSA and 1) the presence and severity of multimorbidity (multiple co-occurring chronic conditions), and 2) subcategories of multimorbidity.

**Methods:**

A cluster sampling technique was used to recruit 120 patients presenting with OSA of various severities from the records of a sleep laboratory in 2008. Severity of OSA was based on the results of the polysomnography. Patients invited to participate received a mail questionnaire including questions on sociodemographic characteristics and the Disease Burden Morbidity Assessment (DBMA). They also consented to give access to their medical records. The DBMA was used to provide an overall multimorbidity score and sub-score of diseases affecting various systems.

**Results:**

Bivariate analysis did not demonstrate an association between OSA and multimorbidity (r = 0.117; *p* = 0.205). However, severe OSA was associated with multimorbidity (adjusted odds ratio = 7.33 [1.67-32.23], *p* = 0.05). OSA was moderately correlated with vascular (r = 0.26, *p* = 0.01) and metabolic syndrome (r = 0.26, *p* = 0.01) multimorbidity sub-scores.

**Conclusions:**

This study showed that severe OSA is associated with severe multimorbidity and sub-scores of multimorbidity. These results do not allow any causal inference. More research is required to confirm these associations. However, primary care providers should be aware of these potential associations and investigate OSA when deemed appropriate.

## Background

Millions of North Americans are affected by the consequences of sleep disorders. Among these disorders, sleep apnea syndrome has the highest rate of mortality and morbidity [1]. According to the Public Health Agency of Canada, 858,900 Canadians reported suffering from sleep apnea, and almost 26% of Canadians are at high risk of developing the condition [2]. This disorder poses a major public health problem due to its prevalence, severity and socioeconomic burden. Obstructive sleep apnea (OSA) is defined as the cessation of naso-buccal air flow for more than 10 seconds [3], and is diagnosed based on an apnea–hypopnea index (AHI) value greater than five per hour of sleep [4], usually accompanied by a 4% decrease in oxygen saturation [4]. It is estimated that 80% of obstructive sleep apnea cases remain undiagnosed [5], making it difficult to identify patients at risk of associated comorbidities [6]. Reuveni et al. suggest that programs be developed to increase the level of suspicion of OSA among primary care providers [7].

OSA syndrome is independently associated with an increased risk of mortality [8,9]. Fletcher [10] reported that 70% to 90% of patients with OSA have hypertension [10]. Associations between OSA and heart failure [11], OSA and arrhythmias [12], OSA and diabetes [13], OSA and insulin resistance [14] and OSA and metabolic syndrome [15] have also been reported. Successful treatment of OSA helps to better control many of the associated diseases and chronic conditions [11,16-18]. Men, people 40 years old and over, and those with a high body mass index (BMI) or a large neck circumference are at greater risk for OSA [19-21].

Multimorbidity—the co-occurrence of two or more chronic diseases—is an emerging concept in the medical literature [22]. One study showed that nine out of ten primary care patients had more than one chronic condition, while approximately 50% had five or more [23]. Multimorbidity has been associated with several adverse effects, such as a reduction in quality of life [24,25], an increase in psychological distress [26], medical complications and increased mortality [27].

Evidence of an association between OSA and multimorbidity could be an important incentive for the systematic screening for OSA in primary care settings—where the prevalence of multimorbidity is very high. The first objective of this study was to measure the association between the severity of OSA and the severity of multimorbidity, and second, to explore the association between OSA and various categories of multimorbidity.

## Methods

This study used data from the sleep laboratory of the Centre de santé et de services sociaux de Chicoutimi (CSSSC), a regional health centre in the Saguenay region of Québec (Canada). As a first step in the recruitment process, a list of patients who had undergone polysomnography in 2008 was compiled. Patients were categorized according to the severity of their OSA, based on their polysomnography results (absent: AHI 0-4; mild: AHI 5-14; moderate: AHI 15-29; severe: AHI ≥ 30). We selected consecutive patients from each category to ensure a proportional representation (25% each) of the four OSA categories. French-speaking patients were selected between 30 and 75 years of age, to ensure adequate variation in degrees of multimorbidity. Each patient underwent polysomnography after January 1, 2008, either in the sleep laboratory as a full night or a split-night study: the first half of the night is used to obtain a diagnosis, the second half is used to perform continuous positive airway pressure (CPAP) titration (level I), or, at home as an outpatient (level II). Patients with a diagnosis of upper airway resistance were excluded from the study, as were people who slept less than three hours a night and those referred for a diagnosis other than apnea, such as parasomnia.

After providing informed consent, participants selected at this stage received a questionnaire covering multimorbidity and socio-demographic variables. Data related to variables of the evaluation conducted at the sleep laboratory were recorded: age, sex, polysomnography results, neck circumference, weight and height.

Several tools are available for measuring multimorbidity. The Disease Burden Morbidity Assessment (DBMA) was selected for this study as it allows to report the absence or presence of 21 predetermined chronic conditions and additional chronic conditions and to determine a functional impact score for each condition on daily life activities [28]. The DBMA is a self-report questionnaire. For each condition present, the patient assesses a degree to which the condition limits his or her activities on a five-point descriptive scale (1: Not at all – 5: A lot). The total score is made up of the sum of all limitations. The metrological qualities and validity of this instrument have been described by Bayliss et al. [28] and a French version was validated in a recent study: sensitivity 73.9% (62.5%-90%); specificity 92.2% (77.6%-98.6%) [29]. The questionnaire was sent by mail based on a modified Dillman method [30]. A second questionnaire was sent to non-respondents 30 days following the first one. Estimated time to complete the questionnaire is approximately 15 minutes.

Due to the exploratory nature of the study, we based our sample size estimation on the availability of the data and feasibility. We aimed for a sample size of 120 (30 per OSA group) to ensure a good representation of each category of the independent variable. We oversampled for a potential non response of 30 to 40%. Consequently, the questionnaire was sent to a convenience sample, as recommended by Dillman [31], of 194 people who had undergone polysomnography at the sleep laboratory of the CSSSC for a diagnosis of sleep apnea in 2008. The study received ethics approval from the Research Ethics Board of the CSSSC.

The subject’s characteristics were described using medians (in the case of asymmetric distributions), means, standard deviations (for continuous variables) and proportions (for categorical variables). A Kolmogorov-Smirnoff test was performed to test for normality of the distributions. In the absence of normality, non-parametric tests were conducted. Bivariate (Spearman rank) correlations were conducted. We dichotomized the AHI by grouping together absent and mild as well as moderate and severe to ensure sufficient size of each group. We performed logistic regression analyses to study the relationship between multimorbidity and OSA. The significance level was set at 0.05, and confidence intervals were calculated at 95%. The DBMA constituted the dependent variable and OSA classification, the independent variable. Other variables were included in the models as adjustment variables (BMI, sociodemographic variables). Neck circumference was not included, as 25% of the data was missing. We dichotomized the DBMA using the median and threshold values of 10 and 20, respectively, to explore the association with clinical variables. Cut-off points were chosen based on the definition of multimorbidity and the results of previous studies [23,32]. A score of 10 means a high impact and at least two chronic conditions; a score of 20 represents a very high impact and at least four chronic conditions (considered here as severe multimorbidity). DBMA sub-groups were formed on the basis of the correlation of each disease with OSA and the conceptual association. We tested three sub-groups: vascular DBMA (hypertension, heart disease, dyslipidemia, heart failure and stroke); cardio DBMA (hypertension, heart disease, dyslipidemia, heart failure) and metabolic syndrome DBMA (hypertension, cholesterol, obesity and diabetes). Data were analyzed using the SPSS package (19.0, SPSS, Chicago. IL).

## Results

Of the 194 patients solicited, seven were non-eligible: five were suffering from parasomnia, one was too old and one we were unable to reach to complete the questionnaire. In total, 187 eligible patients were invited to participate and 120 completed the questionnaire (64.2% response rate). No patients were excluded. Among these, 89.2% of participants had OSA. The average age of patients was 55.5 years, with a predominance of males (65%).

Table 1 presents the characteristics of the 120 patients. The average neck circumference (absent: 41.17 cm; mild: 40.5 cm; moderate: 42.59 cm; severe: 43.99 cm) and BMI average (absent: 32.00; mild: 30.13; moderate: 34.39; severe: 35.77) were higher in moderate and severe OSA.

**Table 1:** Patient characteristics

**Variable**	**AHI**				**Total**
	**Absent**	**Mild**	**Moderate**	**Severe**	
	**(0-4)**	**(5-14)**	**(15-29)**	**(30 and over)**	
Polysomnography results (%)	13 (10.8)	23 (19.2)	36 (30.0)	48 (40.0)	
Neck circumference *	41.17 ± 4.98	40.49 ± 4.12	42.59 ± 5.23	43.99 ± 3.61	42.28 ± 5. 63
Mean ± standard deviation (min and max)	(34.7-50.5)	(31.0-47.0)	(32.0-59.9)	(37.0-51.5)	(31.0-59.9)
Body mass index	32.0 ± 6.7	30.1 ± 6.7	34.4 ± 7.2	35.8 ± 7.7	33.9 ± 7.4
Mean ± standard deviation (min and max)	(22-47)	(18-42)	(24-60)	(21-65)	(18-65)
DBMA	16.0 ± 11.1	14.5 ± 9.6	21.2 ± 11.4	15.3 ±11.8	16.9 ± 11.5
Mean ± standard deviation (min and max)	(2-36)	(0-40)	(2-40)	(1-55)	(0-55)
Mean number of diseases	7 diseases	5 diseases	8 diseases	5 diseases	6 diseases

* N = 90.

The 120 respondents presented six chronic diseases in average. The average number of diseases did not increase in accordance with the severity of OSA (absent: seven diseases; mild: five diseases; moderate: eight diseases; severe: five diseases). Table 2 (DMBA results) shows the distribution of diseases in the sample. The following conditions were present in 50% or more of the patients: obesity (80.8%), hypertension (52.5%) and dyslipidemia (50%).

**Table 2:** DBMA results

**Disease**	**AHI**				**Sampling percentage**
	**0 - 4**	**5 - 14**	**16 - 29**	**(≥ 30)**	
Obesity	10 (8.3%)	17 (14.2%)	31 (25.9%)	39 (32.5%)	97 (80.8%)
Hypertension	6 (5%)	9 (7.5%)	21 (17.5%)	27 (22.5%)	63 (52.5%)
Dyslipidemia	4 (3.3%)	6 (5%)	24 (20%)	26 (21.7%)	60 (50.0%)
Ostheoarthritis	6 (5%)	11 (9.2%)	19 (15.8%)	15 (12.5%)	51 (42.5%)
Back pain or sciatica	6 (5%)	5 (4.2%)	20 (16.7%)	20 (16.7%)	51 (42.5%)
Gastrointestinal ulcer	5 (4.2%)	11 (9.2%)	19 (15.8%)	12 (10%)	47 (39.2%)
Leg circulation problems	4 (3.3%)	7 (5.8%)	18 (15%)	15 (12.5%)	44 (36.7%)
Other joint diseases	5 (4.2%)	6 (5%)	15 (12.5%)	15 (12.5%)	41 (34.2%)
Hearing disorder	1 (0.83%)	8 (6.7%)	11 (9.2%)	18 (15%)	38 (31.7%)
Depression and anxiety	7 (5.8%)	7 (5.8%)	9 (7.5%)	12 (10%)	35 (29.2%)
Diabetes	5 (4.2%)	3 (2.5%)	11 (9.2%)	12 (10%)	31 (25.8%)
Heart disease	2 (1.7%)	5 (4.2%)	9 (7.5%)	14 (11.7%)	30 (25.0%)
Asthma	3 (2.5%)	5 (4.2%)	13 (10.8%)	9 (7.5%)	30 (25.0%)
Vision disorder	1 (0.83%)	2 (1.7%)	9 (7.5%)	7 (5.8%)	19 (15.8%)
Osteoporosis	2 (1.7%)	3 (2.5%)	10 (8.3%)	3 (2.5%)	18 (15%)
Bowel disease	1 (0.8%)	4 (3.3%)	6 (5%)	5 (4.2%)	16 (13.3%)
Chronic bronchitis or emphysema	2 (1.7%)	2 (1.7%)	6 (5%)	5 (4.2%)	15 (12.5%)
Thyroid	1 (0.83%)	2 (1.7%)	4 (3.3%)	5 (4.2%)	12 (10.0%)
Heart failure	2 (1.7%)	0	3 (2.5%)	6 (5%)	11 (9.2%)
Stroke	0	2 (1.7%)	3 (2.5%)	5 (4.2%)	10 (8.3%)
Rheumatoid arthritis	0	0	3(2.5%)	2 (1.7%)	5 (4.2%)
Cancer	0	1 (0.8%)	2 (1.7%)	1 (0.8%)	4 (3.3%)

Table 3 reveals an association between polysomnography results (absent + mild vs. moderate + severe) and BMI (r = 0.261, *p* = 0.01) and gender (r = 0.244, *p* = 0.01) and a similar trend with neck circumference (r = 0.265, *p* = 0.05), and with income (r = 0.218, *p* = 0.05). We were unable to demonstrate a statistically significant association between the DBMA score and dichotomized polysomnography (absent + mild vs. moderate + severe) (r = 0.117, *p* = NS) in this analysis. With regard to multimorbidity sub-scores, Table 3 shows weak correlations between sleep apnea and vascular DBMA and between OSA and metabolic syndrome DBMA. None of the sociodemographic variables were associated with OSA.

**Table 3:** Bivariate analysis with dichotomized polysomnography results

**Variable**	r*	***p *value**
BMI	0.261	0.01
Neck circumference	0.265	0.05
Sex	0.244	0.01
Income	0.218	0.05
Vascular DBMA (sub-score)	0.261	0.01
Cardiac DBMA (sub-score)	0.196	0.05
Metabolic syndrome DBMA (sub-score)	0.261	0.01
DBMA score	0.117	0.205
Age	0.132	0.151
Marital status	0.109	0.090
Place of birth	0.085	0.355
Education	-0.026	0.780

Dichotomized polysomnography: 0 = absent + mild; 1 = moderate + severe

DBMA sub-score: Cardiac DBMA: Hypertension, heart disease, dyslipidemia, heart failure; Vascular DBMA: Hypertension, heart disease, dyslipidemia, heart failure, stroke;

Metabolic syndrome DBMA: Hypertension, cholesterol, obesity and diabetes

*Spearman correlation.

Tables 4 and 5 show a significant association between severity of DBMA and severe OSA. There was also a significant association between DBMA and severe OSA (an AHI over 30). This association was not observed in patients with mild to moderate OSA. None of the sub-scores presented in Table 3 showed an association with severity of OSA in logistic regression analyses (results not shown).

**Table 4:** Logistic regressions unadjusted

**Variable/Result**	**OR**	***p *value**	**95% CI**
Median DBMA (14.00)	Mild: 1.31	0.670	0.38-4.50
	Moderate: 1.17	0.755	0.49-3.21
	Severe: 2.70	0.029	1.11-6.60
DBMA 10	Mild: 1.23	0.755	0.33-4.61
	Moderate: 0.85	0.761	0.31-2.38
	Severe: 3.40	0.031	1.15-10.36
DBMA 20	Mild: 1.33	0. 675	0.35-5.13
	Moderate: 0.63	0.475	0.18-2.23
	Severe: 3.00	0.020	1.19-7.56

Median DBMA: 0 = DBMA from 0 to 13.99 and 1 = 14 and over.

DBMA 10: 0 = DBMA from 0 to 9.99 and 1 = 10 and over.

DBMA 20: 0 = DBMA from 0 to 19.99 and 1 = 20 and over.

**Table 5:** Logistic regression analyses adjusted for sex, age, BMI and income

**Variable/result**	**OR**	***p *value**	**95% CI**
Median DBMA (14.00)	Mild: 0.90	0.909	0.16-5.03
	Moderate: 0.92	0.912	0.22-3.84
	Severe: 3.94	0.021	1.24-12.59
DBMA 10	Mild: 1.41	0.696	0.25-7.92
	Moderate: 0.83	0.798	0.26-3.37
	Severe: 4.34	0.021	1.22-15.44
DBMA 20	Mild: 1.12	0.911	0.16-7.75
	Moderate: 0.32	0.229	0.05-2.07
	Severe: 7.33	0.008	1.67-32.23

Median DBMA: 0 = DBMA from 0 to 13.99 and 1 = 14 and over.

DBMA 10: 0 = DBMA from 0 to 9.99 and 1 = 10 and over.

DBMA 20: 0 = DBMA from 0 to 19.99 and 1 = 20 and over.

## Discussion

The present study revealed an association between severe OSA and severity of multimorbidity as measured by the DBMA. The relationship was still present after adjusting for several potential confounders.

These findings have implications for general practice. Many patients seen in primary care practices present with multimorbidity. In order to investigate the potential association between multimorbidity and OSA, screening could be done clinically or by using a tool such as the Epworth Instrument which is highly correlated with OSA [33]. Patients could be referred to a sleep lab for evaluation when OSA is suspected. If OSA is confirmed, it may affect the management of the patient who could benefit from treatment that has been demonstrated to help control many associated diseases and chronic conditions [11,16-18].

One previous study suggests that multimorbidity exists in OSA [34]. To our knowledge, this is the first study to report an association between severe OSA and multimorbidity. We searched for an exposure–response relationship between OSA and multimorbidity but the composition of our sample prevented us from observing such a relationship. In our sample we did not obtain the desired proportion representation (25% each) of the four severity categories of the independent variable (OSA: absent, mild, moderate and severe). We sent the DBMA questionnaire to 50 people in each OSA classification category but we had a disproportionately high response rate in the severe category (48 out of the 50 patients) compared to the others. On the other hand, we obtained a sample of relatively sicker subjects (an average DBMA of 16). Patients in this study presented more chronic conditions (six per person) than reported by Fortin [23] (4.6 per person) or Kadam (1.3 per person) [32] in their assessment of multimorbidity in primary care practices. We suspect that the disproportionate response rate is due to the fact that patients who were sicker were more interested in participating in this study.

Regarding other characteristics and associations, the respondents were fairly representative of the sleep apnea population, with a predominance of male subjects [21]. Neck circumference and BMI were found to be positively associated with OSA, which was expected [21]. We observed an association between the AHI and metabolic syndrome. This association had been previously found by other groups [35,36]. Although not addressed in our study, associations were also found between OSA and each component of the metabolic syndrome. In fact, the evidence suggests that OSA is actually part of the metabolic syndrome. One study suggests that OSA symptoms (snoring, hyper somnolence) predict the development of metabolic syndrome. In addition, evaluation of OSA symptoms can help identify individuals who are at risk of developing metabolic syndrome [35].

Our sleep laboratory uses a definition of hypopnea which is accepted in the literature: a decrease in respiratory amplitude of 50% or more, accompanied by a desaturation of 3% [37]. However, other laboratories require a 4% desaturation for a positive diagnosis. The method used in this study is therefore more sensitive than the diagnostic criteria used in some laboratories. This could result in higher AHI values and the number of diagnoses of patients with mild or moderate symptoms who would not have been diagnosed by other laboratories. Several epidemiological studies have established a relationship between OSA and vascular morbidity using the 4% cut-off point [38]. This may explain why we did not observe any link between our groups of mild or moderate subjects and a measure of morbidity. It is especially important that studies showing a link between sleep apnea and morbidity examine these associations, particularly in the case of severe OSA (AHI greater than 30). Also, if OSA is an intermediate factor in the development of hypertension, diabetes, dyslipidemia or other conditions, controlling for these variables represents a case of "over adjustment," potentially affecting the association between dependent and independent variables [39,40].

For more than 20 years, cross-sectional studies, control cases and other evidence have suggested an association between OSA and heart disease, heart failure, arrhythmias and cerebrovascular diseases [8,9,12,40]. A gradation of vascular risk in relation to AHI has been proposed [40]. We observed an association between vascular DBMA and AHI when AHI was dichotomized into absent and mild versus moderate and severe. Similar results were obtained in prospective population studies, such as the Sleep Heart Health Study [41] and the Wisconsin Sleep Cohort Study [42]. In the first study, Gottlieb identified an association between incident heart failure and OSA. Although there is no defined threshold value, this association is especially important in subjects with an AHI greater than 30. In the Wisconsin study, the associations were significant only in subjects with an AHI greater than 30 (odds ratios of 4.5 and 5.2, respectively, for risks of cerebral vascular disease and cardiovascular death). Furthermore, a longitudinal study showed that men are at increased risk of stroke when the AHI is greater than 19 per hour of sleep. Among women, unadjusted overall results showed an increased risk threshold at 25 AHI per hour of sleep (*p* = 0.002) [39].

This study has limitations. With a limited response rate of 64%, we were unable to obtain a proportional representation in categories of OSA classification in our sample, and this may have confounded the relationship between the DBMA and OSA. In addition, we had to remove one variable from the analysis (neck circumference) due to a high number of missing values. Another limitation of the study is the use of the DBMA as a measure of multimorbidity. The DBMA measures self-reported disease burden that correlates well with quality of life outcomes [28]. However, in other multimorbidity measures, disease severity is evaluated based on purely clinical criteria assessed by health professionals. The different methods of evaluating disease severity may have an impact on the association between multimorbidity and OSA.

## Conclusions

In this study we found a link between severe obstructive sleep apnea and severity of multimorbidity. These results represent the first documentation of a relationship between severity of OSA and severity of multimorbidity. The study also showed an association between OSA and multimorbidity sub-scores (cardiac, vascular, metabolic syndrome). Primary care providers should be aware of these potential associations and investigate OSA when deemed appropriate. There is a need for additional research in this area, and our findings may help raise awareness among family physicians about this condition and improve access to diagnosis and treatment. Research would benefit from repeating the same study using a longitudinal study design.

## Competing interests

This was not an industry-supported study and the authors have indicated no financial conflict of interest. This research received financial supported from the CIHR Applied Research Chair – Health Services and Policy Research on Chronic Diseases in Primary Care/Canadian Institutes of Health Research-Institute of Health Services and Policy Research, Canadian Health Services Research Foundation and Centre de santé et de services sociaux de Chicoutimi.

## Authors’ contributions

LRH conceived the study, conducted the data collection, and participated in its design and in the data analysis. LRH also wrote the manuscript. MB and MF supervised the first author’s work and participated in its design and data analysis. All authors read and approved the final manuscript. LRH takes responsibility for the integrity of the work as a whole.

## Pre-publication history

The pre-publication history for this paper can be accessed here:

http://www.biomedcentral.com/1471-2466/12/60/prepub
